# Editorial: Frailty- and age-associated diseases: possibilities for intervention

**DOI:** 10.3389/fragi.2024.1522451

**Published:** 2024-11-28

**Authors:** Cristina Mas-Bargues, Jose Viña, Consuelo Borrás

**Affiliations:** Freshage Research Group, Department of Physiology, Faculty of Medicine, University of Valencia, Institute of Health Research-INCLIVA, and Centro de Investigación Biomédica En Red: Fragilidad y Envejecimiento Saludable (CIBERFES), Valencia, Spain

**Keywords:** frailty, cellular senescence, exercise intervention, early detection, polypharmacy

## Introduction

Frailty, a complex and multifaceted syndrome characterized by reduced strength, endurance, and physiological function, has gained significant attention in recent years due to its profound impact on the aging population. As the global population ages, the prevalence of frailty and age-associated diseases has become a pressing public health concern. Frailty is characterized by a decline in physiological reserve and increased vulnerability to stressors, often followed by a cascade of health issues, including musculoskeletal disorders, cardiovascular diseases, metabolic alterations, and neurodegenerative conditions. These interconnected maladies significantly compromise the quality of life for older adults, posing challenges for healthcare systems and society at large. Central to the development of frailty and these diseases is the phenomenon of cellular senescence. This process, wherein cells enter a state of permanent growth arrest in response to stressors, such as oxidative damage and inflammation, contributes to the aging process and the onset of age-related pathologies. Senescent cells secrete pro-inflammatory factors, known as the senescence-associated secretory phenotype (SASP), which can disrupt tissue homeostasis and exacerbate chronic conditions. Consequently, targeting cellular senescence presents a promising avenue for intervention, potentially reversing or mitigating the impacts of frailty and associated diseases (Mylonas and O’Loghlen). This editorial synthesizes the insights from ten articles to establish the main hallmarks of current research and interventions in frailty management, highlighting the multidimensional strategies necessary for addressing frailty in older adults. The evidence from these articles collectively points toward a comprehensive approach that integrates exercise and rehabilitation, early detection, medication management, inflammation control, and individualized care.

### The central role of exercise and rehabilitation in frailty reversal

One of the most consistent and well-supported interventions across the articles is the role of exercise in mitigating frailty and improving physical outcomes in older adults. Physical frailty is often associated with conditions such as sarcopenia, cognitive decline, and general mobility impairment, all of which can significantly impair an individual’s independence and quality of life. Exercise-based interventions, particularly those involving multi-component programs, have the potential to reverse or delay the progression of frailty.

For example, structured physical activity programs that incorporate resistance training, balance exercises, and aerobic activities have shown significant benefits in reducing frailty scores, improving muscle mass, and enhancing physical function. Importantly, these interventions are often tailored to the specific needs of older adults, accounting for their capabilities and limitations. Rehabilitation programs designed for those recovering from strokes or other acute illnesses also demonstrate positive outcomes, highlighting the adaptability of exercise interventions across various medical conditions (Luo et al.).

One of the key benefits of exercise in frailty management is its ability to target multiple physiological systems. Resistance training, for instance, enhances muscle strength and function, addressing the muscle wasting commonly seen in sarcopenic individuals. Balance and mobility exercises, on the other hand, reduce the risk of falls, a major concern for frail older adults. Moreover, cardiovascular fitness improvements achieved through aerobic exercises help combat fatigue and increase overall endurance, providing a global approach to improving physical resilience.

### The critical importance of early detection and screening

Frailty is often underdiagnosed or detected at advanced stages, when interventions may be less effective. Several articles emphasize the importance of early detection tools and screening methods in identifying frailty and related conditions, such as sarcopenia and osteoporosis, before they lead to debilitating consequences. This proactive approach to frailty management is crucial, as it allows healthcare providers to intervene before the condition progresses to a point where it severely compromises an individual’s quality of life.

Among the screening tools mentioned, the SARC-F questionnaire is a widely recognized and practical instrument for assessing sarcopenia, one of the key contributors to physical frailty. The SARC-F assesses key aspects of muscle function, such as strength, assistance with walking, and difficulty standing from a chair. This tool has proven useful in both clinical settings and population-level screenings for identifying individuals at risk of muscle deterioration (Nguyen et al.; Valencia-Muntala et al.). Dual-energy X-ray absorptiometry (DXA) scans, commonly used to assess bone density, are also important for diagnosing osteoporosis, a condition closely linked to frailty due to the increased risk of fractures. Early identification of osteoporosis allows for timely interventions (Jianu et al.). Moreover, the competing risk nomogram developed for older patients with cancer emphasizes the value of predictive modeling in clinical practice. By identifying high-risk patients and intervening early, clinicians can enhance prognostic accuracy and guide treatment plans, ultimately improving patient outcomes (Wu et al.).

The integration of screening tools for sarcopenia, osteoporosis, and cancer into standard geriatric care is critical for preventing the downward spiral of frailty.

### Medication management and poly-deprescribing as key interventions

Polypharmacy, or the concurrent use of multiple medications, is another significant concern in the management of frailty and age-associated diseases. Many older adults are prescribed a range of medications to manage chronic conditions such as hypertension, diabetes, and cardiovascular diseases. However, the simultaneous use of multiple drugs can lead to adverse drug reactions, cognitive impairment, and decreased physical function, all of which contribute to frailty.

A key finding across the articles is the importance of poly-deprescribing, a process that involves the careful reduction or discontinuation of unnecessary medications in older adults. Deprescribing strategies have been shown to improve outcomes such as cognitive function, reduce the risk of falls, and enhance overall wellbeing. This process must be handled with care, as abrupt cessation of essential medications can have negative consequences. Therefore, the deprescribing process should involve a comprehensive review of each patient’s medications, with a focus on maintaining the most beneficial treatments while eliminating those that contribute to frailty or increase the risk of adverse events (Garfinkel and Levy).

Also, the use of conventional, herb-based medicine can be a potential therapeutic intervention for frailty but also a preventive method (Amitani et al.) for mild and moderate frailty.

### The role of inflammation and comorbidity management in frailty

Frailty is closely linked to chronic inflammation and the presence of multiple comorbidities, such as diabetes, cardiovascular disease, and neurodegenerative conditions like Parkinson’s disease. Chronic low-grade inflammation, often referred to as “inflammaging,” is a hallmark of aging and has been implicated in the development of frailty. Inflammation contributes to muscle wasting, cognitive decline, and increased vulnerability to infections and other stressors, all of which can accelerate frailty.

Addressing the inflammatory burden in frail individuals is, therefore, a crucial component of frailty management. Anti-inflammatory interventions, whether through pharmacological agents or lifestyle modifications such as diet and exercise, can help reduce the impact of systemic inflammation on physical and cognitive function. Some studies have explored the use of anti-inflammatory drugs, such as nonsteroidal anti-inflammatory drugs (NSAIDs) or more targeted therapies, to reduce frailty in older adults, although more research is needed to determine the long-term efficacy and safety of these approaches (Merchant et al.).

Additionally, managing comorbid conditions that contribute to inflammation and frailty is essential for improving outcomes in older adults. For example, optimal management of diabetes through glycemic control can prevent complications such as neuropathy and cardiovascular disease, both of which exacerbate frailty (Komici et al.). By taking a synergistic approach to inflammation and comorbidity management, healthcare providers can help mitigate the impact of these factors on frailty progression.

## Conclusion

The management of frailty and age-associated diseases requires a comprehensive, multifactorial approach that integrates exercise, early detection, medication optimization and inflammation control, and individualized care. The findings from these ten articles highlight the importance of targeting both the physical and cognitive components of frailty, addressing underlying comorbidities, and tailoring interventions to each individual’s unique needs. By adopting a proactive and personalized approach to frailty management, healthcare providers can improve outcomes for older adults and help them maintain independence and quality of life as they age. The future of frailty research and clinical care lies in further refining these strategies to create a robust framework for managing frailty in an increasingly aging population.

